# Mental health in children of parents being treated by specialised psychiatric services

**DOI:** 10.1177/14034948221076208

**Published:** 2022-02-22

**Authors:** Emme-Lina W. Nordh, Gisela Priebe, Karin Grip, Maria Afzelius, Ulf Axberg

**Affiliations:** 1 Department of Psychology, University of Gothenburg, Sweden; 2 Department of Social and Psychological Studies, Karlstad University, Sweden; 3 Department of Social Work, Malmo University, Sweden; 4 Faculty of Social Studies, VID Specialized University, Norway

**Keywords:** Parental mental illness, specialised adult psychiatry, children at risk, child mental health, family context, cumulative risk

## Abstract

**Background:**

One in ten children have a parent diagnosed with a mental illness by specialised psychiatric services. Severe parental mental illness is a well-established risk factor for children’s mental health problems, making the identification and support of these children a public health concern. This study investigated the mental health and family context of children of parents diagnosed with depression, anxiety, or bipolar disorder in this clinical setting.

**Methods:**

Parental reports on 87 children aged 8–17 years were analysed. The children’s mental health was compared with that of a Swedish population-based sample. Multiple linear regression was used to investigate associations between child mental health and child gender, child age, parent symptoms and social status, family functioning, and perceived parental control. Furthermore, a cumulative risk index explored the effect of multiple risk factors on child mental health.

**Results:**

The children reportedly had significantly more mental health problems than did the population-based sample and about one-third had scores above the clinical cut-off. A significant multiple linear regression explained 49% of the variance in child mental health, with lower perceived parental control and younger child age being associated with more child mental health problems. With more reported risk factors, children reportedly had more mental health problems.

**Conclusions:**

**The results underline the importance of identifying a patient’s children and assessing multiple relevant risk factors in the child’s life. Furthermore, the results indicate that the needs of younger children and of patients in their parenting role are important to address.**

## Background

It has been estimated that about one in four children under 16 years of age have a mother with a mental illness [1] and that about one in ten children under 18 years of age have a parent with a severe mental illness diagnosed by specialised psychiatric services [2]. It is well established that children of parents with mental illness have an increased risk of developing their own mental health problems [3] and they also have an increased risk of experiencing other negative outcomes, such as poor physical health [4] and difficulties with academic performance [5]. Identifying and adequately supporting children of parents with mental illness is therefore important from a public health perspective [1–3]. Specialised adult psychiatric services have a key role in identifying these children [3,6], and a few countries, including Sweden [7], have introduced laws to ensure that their needs are addressed in the health-care system. However, research has indicated that not all children of patients treated by specialised psychiatric services are identified [6,8] and that only a small part are given preventive interventions or are involved in collaboration with other agencies [6].

Previous international research has found that children of parents with mental illness and in contact with psychiatry or welfare services reportedly have more mental health problems than do children not living with parents with mental illness [9]. The objective of this study was to investigate the mental health of children of Swedish psychiatric patients, and to investigate relevant risk factors that can be assessed and addressed in clinical practice. The study is part of a research project supported by the Swedish National Board of Health and Welfare, evaluating preventive interventions for 8–17-year-old children of parents diagnosed with depression, anxiety or bipolar disorder by specialised adult psychiatry. The age span and diagnoses were chosen in view of the target group of the included preventive interventions. Furthermore, depression and anxiety disorders are common mental illnesses in psychiatric patients who are parents [6,10].

Whether and how a child is negatively affected by parental mental illness depends on biological, psychological, and social risk and protective factors in the child, parent, family and community [11,12]. Besides genetic vulnerability and prenatal influences, important risk factors concern how the mental illness affects the parent’s cognition, emotions, and behaviour, parent–child interaction, and the family environment [11,12]. Parents’ self-efficacy beliefs concerning their ability to influence their child is a factor linked to child and parent well-being and parent–child interaction [13]. Perceived parental control is a concept arguably close to parental self-efficacy beliefs [14], and low perceived parental control of troublesome child behaviours as well as low parental self-efficacy beliefs are associated with both internalising and externalising problems in children [13,14]. Family functioning refers to the collective health of the whole family, and family dysfunction is reported in many families experiencing parental mental illness [15,16], which has been associated with depressive symptoms in children of parents with depression [16]. Several other factors are relevant when assessing risk exposure in this group of children. The type of parental mental illness and its characteristics influence how children are affected, and the risk of negative outcome is increased for children of parents with severe or recurrent mental illnesses [11]. Other more general risk factors for mental illness, such as parental unemployment, socioeconomic disadvantage, and single parenthood, are common in these families [2]. With more risk factors present, the risk of a negative outcome has been found to increase for the child [17,18].

## Aims

The aim of this study was to investigate child mental health and family context in 8–17-year-old children of parents being treated for depression, anxiety or bipolar disorder by specialised psychiatric services. More specifically, the aims were to (a) compare parent-reported child mental health with that in a population-based sample; (b) investigate the associations between parental mental health, family functioning, perceived parental control, sociodemographic characteristics, and child mental health; and (c) investigate whether perceived parental control, family functioning, parental mental health, and sociodemographic characteristics could predict child mental health. Furthermore, the aim was to (d) explore the number of reported risk factors present in the life of the child and their associations with child mental health.

## Methods

### Procedure

This study was conducted during clinical practice in specialised psychiatric services for adults and used baseline data from a longitudinal research project investigating preventive interventions for children aged 8–17 years, given as part of the patient’s normal treatment process. Receiving a preventive intervention did not include extra fees beyond the patient’s regular treatment fee.

A member of the research team informed all mental health professionals from 46 psychiatry units, located in five regions in Sweden, about the project. Professionals who, during the inclusion period (September 2014 to December 2017), initiated support targeting a patient’s children, either directly or indirectly, first gave verbal and written information about the study to the patient without the partner or children present. If the patient consented, the partner was informed and asked to participate. Written informed consent was obtained from all participants. At the beginning of the intervention, participants individually completed a questionnaire, either on paper or online, at the psychiatry unit or at home, and couples were asked not to discuss the questions with each other. If the participants had more than one child aged 8–17 years, they were asked to answer questions about each child. It was estimated to take about 30 min to complete the questionnaire. The participants could pause and return to the questions later if needed.

Parents were chosen as informants so as to include information about as many children as possible, as the youngest children (aged 8–9 years) were not old enough to independently complete the questionnaires. It was also expected that not all patients would want their children to participate, as research indicates that not all children know about their parents’ contact with psychiatry [10]. There is a risk of biased responses in reports by parents with mental illness, towards reporting more mental health problems in their children than is evident in child self-reports [19]. However, in families in which there is parental depression, it has also been shown that parental reports and child self-reports were equally good in predicting onset of depression in the children, and that parental reports were better in predicting new onset of depression in younger children [19].

### Participants

**Figure 1.**
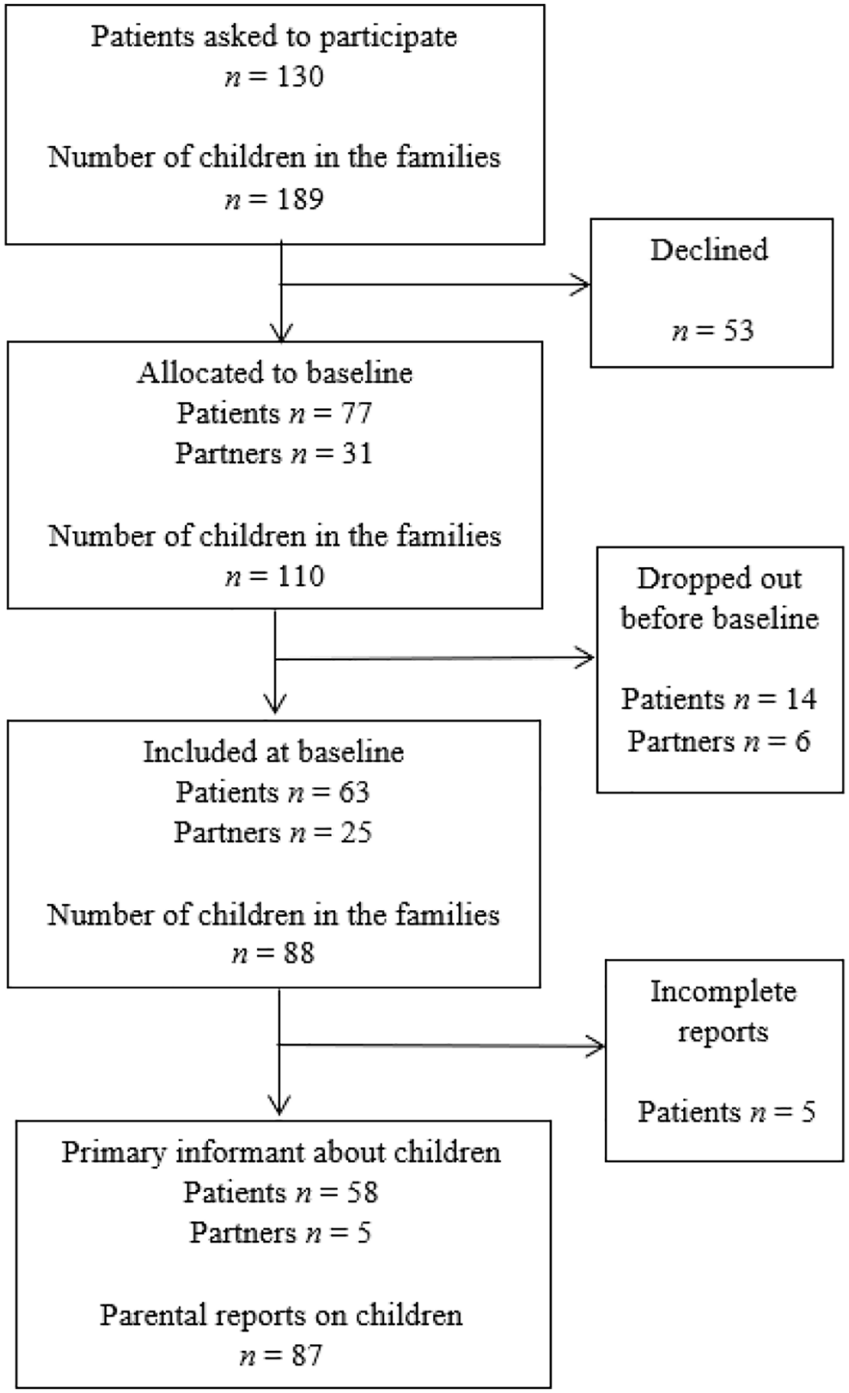
Participant inclusion process.

During the inclusion period, 130 patients were informed about the study and asked to participate; 60 (46%) of them completed the questionnaire, as did a group of their partners (*n* = 25). The participants were recruited from 16 of the participating psychiatry units (15 outpatient and one inpatient). Parental reports about 87 children from 63 families were used in the analyses (see inclusion process in Figure 1).

The inclusion criteria were patients diagnosed with depressive, bipolar and/or anxiety disorder being treated by specialised psychiatric services, about to receive a preventive intervention with a focus on their children aged 8–17 years. Sufficient knowledge of Swedish to answer the questions independently was required to participate. Exclusion criteria were if the patient, during the past 12 months, had previously received a preventive intervention from specialised psychiatric services, with a focus on their children aged 8–17 years; if the patient had a main diagnosis of substance use or schizophrenia; or if the family was experiencing an ongoing severe crisis, for example, violence or a recent death in the family. Parental reports were not collected for children in treatment for depression or anxiety disorder. This exclusion criterion was imposed given the aim of the longitudinal project, which was to evaluate the effectiveness of the preventive interventions.

### Ethical considerations

The Regional Ethics Committee in Gothenburg approved the research project (reg. no. 1029-13). The mental health professionals made the decisions to offer the preventive interventions and to ask patients to participate in the research project. The preventive interventions included in the project were not intended to be used in acute phases of parental mental illness or if a family crisis was ongoing, as stated in the exclusion criteria. Steps were taken to ensure that the participants could consult the research team or the mental health professionals with questions or concerns. Only the research team had access to the questionnaire responses.

### Measures

The *Strength and Difficulties Questionnaire – Parent version* (SDQ-P*)* [20,21] was used to measure child mental health problems. The measure includes 25 items about child behaviours and psychological attributes such as “Often unhappy, down-hearted or tearful” and “Nervous or clingy in new situations, easily loses confidence”, which are responded to on a three-point scale. The measure consists of five subscales, that is, Emotional Symptoms, Conduct Problems, Hyperactivity–Inattention, Peer Problems, and Prosocial Behaviour. All but the Prosocial Behaviour subscale can be summed into a Total Difficulties score (range 0–40), with higher scores indicating more difficulties. A brief Impact Supplement asks whether the respondent thinks the child has a problem and about overall distress and social impairment. Swedish clinical cut-off scores [21] and a Swedish population-based sample of children aged 10–13 years (*n* = 437) [20] were used for comparison. The Total Difficulties score was the primary outcome measure in this study, for which Cronbach’s α was .81. For the subscales and the Impact Supplement, α varied between .70 and .78, except for the Conduct Problems subscale, for which α was .53, which must be noted when interpreting the results for this scale.

The *Clinical Outcomes in Routine Evaluation – Outcomes Measure* (CORE-OM) [22] was used to measure distress in parents. The measure comprises 34 items covering four conceptual domains: Well-being, Symptoms (anxiety, depression, or physical), Functioning (close relations, general, or social), and Risk to Self and/or Others. Items, for example, “I have felt totally lacking in energy or enthusiasm”, are responded to on a five-point scale. The items can be summed into a Total Scale, either including or excluding Risk items, with higher scores indicating more distress. Swedish clinical cut-off scores [22] were used. The Total Scale excluding Risk items, for which α was .96, and the Symptoms subscale, for which α was .93, were used in the analyses.

The *Hospital Anxiety and Depression Scale* (HADS) [23] was used to identify self-reported symptoms of depression and anxiety in parents. The scale comprises 14 items, divided into two subscales, and items such as “Worrying thoughts go through my mind” are rated on a four-point scale. A cut-off score of 11 or above on the subscales indicates the probable presence of symptoms at a clinical level, and participants with scores of 8–10 are considered possible cases. In this study, α was .88 for both subscales.

The *Family Assessment Device* (FAD*)* [24] is a 60-item measure of the respondent’s perception of different dimensions of family functioning. The 12-item General Functioning subscale (FAD-GF) can be used on its own to assess the overall functioning and emotional health of the family through items such as “There are lots of bad feelings in our family”, which are rated on a four-point scale, with higher scores representing more problematic functioning. Cut-off scores for problematic family functioning from the original study [24] were used. Cronbach’s α was .87 for the GF subscale, which was used in the analyses.

The subscale *Perceived Parental Control of Child’s Behaviour* (PLOC-PPC) [14] of the Parental Locus of Control Questionnaire [25] was used to measure perceived parental control in rearing situations, perceptions proposed to be related to parental self-efficacy beliefs [14]. The subscale consists of 10 personalised statements about the parent’s perception of being in control of troublesome child behaviours, for example “My child’s behaviour is sometimes more than I can handle”. The items are responded to on a five-point scale, with higher scores indicating more perceived control. A general population sample of Swedish children (*n* = 70, age 9 years) was used for comparison [14]. The Cronbach’s α of PLOC-PPC was .83.

*Sociodemographic questions* covered age, gender, country of origin, civil status, and number of children. The Hollingshead Index of Social Status (range 8–66) was used, according to which participants could fall into a low (<30) or average (⩾30) category [26]. A reference group of Swedish parents [27] coming with their children (aged 8–19 years) to a routine examination at a public dental clinic was used for comparison. Questions about children concerned legal custody, residence arrangements, and contact with Child and Adolescent Mental Health Services (CAMHS). Patients were asked about the length of contact with psychiatric services. The mental health professionals reported basic sociodemographic information and the reason for contact with psychiatry for all patients they had asked to participate.

A *cumulative risk index* was constructed in which child, parent and family risk factors documented in previous research as well as variables found to predict child mental health in this study were included. The presence of a risk factor was coded as 1 if present and 0 if absent, and values for all risk factors were summed into a total risk score (range 0–6). The following variables were included and coded as 1 if present: young child age (8–10 years), low social status of parent, single parenthood, parent score above the clinical cut-off on the CORE-OM Symptoms subscale, long contact with specialised psychiatric services (top 25th percentile in our sample), and low perceived parental control (below the 25th percentile in our sample).

### Statistical analyses

The Pearson’s chi-squared (χ^2^) and Fisher’s exact tests were used to analyse differences in categorical variables. Independent-sample *t*-tests were carried out to assess differences in continuous variables. Pearson’s correlations between study variables were calculated. A multiple linear regression was calculated to predict the dependent variable child mental health (SDQ-P Total Difficulties score) based on the independent variables FAD-GF and PLOC-PPC, controlling for child age and gender, parents’ Social Status (SS), and parental anxiety (HADS-A). The assumptions for the multiple regression analysis were examined and found to be met. The independent variables were entered simultaneously by forced entry, as the order of variables was not predetermined. To explore the effect of the number of risk factors on child mental health, the mean SDQ-P Total Difficulties scores were compared between subgroups in this sample, that is, experiencing 0–1, 2–3 or 4–6 risk factors, and the population-based sample. For all tests, *p* <.05 was considered significant and the effect sizes for mean comparisons were computed using Cohen’s *d*, with definitions of small (*d* = 0.20), medium (*d* = 0.50), and large (*d* = 0.80) [28].

In the analyses based on parental reports on children, data from one primary informant for each child were used. For 81 children, the patient was the primary informant (*n* = 58), and for six children, when the patient’s data were missing or incomplete, the patient’s partner was the primary informant (*n* = 5). To control for possible differences between patient and partner ratings in our sample, we compared ratings in families in which both had rated the same children (*n* = 33), and no significant difference in the SDQ-P Total Difficulties score was found.

The percentages of items missing from the included standardised measures were 0.5–4.8%. When summed into scale scores, missing values were handled according to scale guidelines, either being replaced with the participant subscale mean or not calculated, depending on how many items were missing. There was no scale guideline for PLOC-PPC, so the authors decided that two items could be missing when calculating a scale score. The data were examined before analyses and no extreme outliers were found, and the normality assumption was judged to be fulfilled.

Statistical analyses were conducted using IBM SPSS Statistics version 26.0 (IBM, Armonk, NY, USA) and Laken’s Excel sheet version 4.2 [29].

### Attrition analysis

When comparing the patients in the participating families (*n* = 63) with those who chose not to participate (*n* = 67), it was found that significantly more patients with bipolar disorder as the reason for contact with psychiatry chose to participate (*χ*^2^ (1) = 4.61, *p* =.04). No other differences were found concerning basic background information. Concerning the children, no significant differences concerning child age or gender were found between children in participating and non-participating families.

## Results

### Description of sample

The reasons for contact with psychiatry were depression for 24 patients (39%), anxiety for eight (13%), both depression and anxiety for five (8%), and bipolar disorder for 25 (40%). The patients’ current contact with specialised psychiatric services had lasted 1–12 months for 15 patients (27%), >1–2 years for 16 (29%), 3–6 years for 10 (18%), 7–10 years for six (11%), and over 10 years for nine (16%) patients.

The sociodemographic characteristics of the parent-rated children (*n* = 87) and the primary informants (patients *n* = 58, partners *n* = 5) are found in Table I. The SS of the primary informants was significantly lower, that is, *t*(284) = 5.11, *p* < .001, *d* = 0.74, 95% CI [0.45, 1.03], than that of the reference group [27] (*n* = 226, *M* = 38.2, *SD* = 11.7).

**Table I.** Characteristics of parent-rated children and primary informants.

Characteristic	Parent-rated children	Primary informants
Age, *M* (*SD*), years	11.9 (2.8)	41.0 (7.2)
Gender, *n* (%)		
Female	36 (41)	43 (68)
Male	51 (59)	20 (32)
Country of origin, *n* (%)		
Sweden	84 (97)	53 (84)
Nordic countries	2 (2)	1 (2)
Europe	1 (1)	2 (3)
Outside Europe	0 (0)	5 (8)
Missing	0 (0)	2 (3)
Parent who is the patient, *n* (%)		
Mother	55 (63)	
Father	32 (37)	
Custody, *n* (%)		
Joint^†^	81 (93)	
Sole	4 (5)	
Other	2 (2)	
Residence arrangements, *n* (%)		
Patient and partner	48 (55)	
Dual residence^‡^	28 (32)	
Primarily with patient	8 (9)	
Primarily with partner	2 (2)	
Only with patient	1 (1)	
Contact with CAMHS^§^, *n* (%)		
Yes	17 (20)	
No	70 (80)	
When in contact with CAMHS, *n* (%)		
During past 6 months	5 (29)	
7–18 months ago	7 (41)	
Further back in time than 18 months	5 (29)	
Number of children <18 years in household, *M* (*SD*)		2.1 (0.9)
SS^¶^, *M* (*SD*)		29.0 (14.8)
SS Low (<30), *n* (%)		37 (59)
SS Average (⩾30), *n* (%)		26 (41)
Civil status, *n* (%)		
Married or living together		38 (60)
In a relationship, but not living together		7 (11)
Single		16 (25)
Missing		2 (3)

*Note*: Parent-rated children, *n* = 87, and primary informants, *n* = 63, except for Age, *n* = 62, and SS, *n* = 60. For residence arrangements, civil status, and when in contact with CAMHS, the sum of percentages is out by 1% due to rounding.

† Joint custody = parents have shared decision-making. ^‡^Dual residence = child lives equal amounts of time with both separated parents. ^§^CAMHS = Child and Adolescent Mental Health Service. ^¶^SS = social status according to the Hollingshead Index.

### Parent-reported child mental health

According to parent-reported SDQ-P results, the children had significantly more mental health problems in relation to the problem subscales and the Total Difficulties scale than did the population-based sample [20], and the effect sizes of the difference were between small and medium (see Table II). Relative to Swedish clinical cut-off scores [21], 34% of the children exceeded the cut-off for the Total Difficulties scale. When the Total Difficulties scale was combined with the Impact score, 18 children (21%) had scores above the clinical cut-off for both, indicating clinical-level symptoms interfering with the daily life of the child.

**Table II.** Parent-rated child mental health (SDQ-P^†^) results in this study, compared with a population-based sample [20] and relative to clinical cut-off scores [21].

Subscale	*Study sample*		*Population-based sample*		*p*	*d*	95% CI	*Clinical cut-off*	
	*M*	*SD*	*M*	*SD*				Score	*n (%)*
Emotional Symptoms	2.83	2.60	1.7	1.80	<.001***	0.58	[0.34, 0.81]	⩾3	40 (47)
Conduct Problems^‡^	1.53	1.56	1.0	1.2	<.001***	0.42	[0.19, 0.65]	⩾3	22 (26)
Hyperactivity–Inattention	3.01	2.27	2.3	2.1	.005**	0.33	[0.10, 0.57]	⩾4	31 (36)
Peer Problems	1.69	1.86	1.2	1.5	.008**	0.31	[0.08, 0.55]	⩾2	41 (48)
Prosocial Behaviour	8.10	1.89	8.3	1.7	.328	0.12	[−0.12, 0.35]	⩽7	16 (19)
Impact Supplement	0.74	1.52	–	–	–	–	–	⩾1	25 (29)
Total Difficulties	9.06	5.80	6.2	4.7	<.001***	0.58	[0.35, 0.82]	⩾11	29 (34)

*Note*: Study sample, *n* = 86; population-based sample, *n* = 437. CI = confidence interval; - = no Swedish reference population available for comparison.

† SDQ-P = Strength and Difficulties Questionnaire – Parent version. ^‡^The results must be interpreted with caution due to the low Cronbach’s alpha of .53.

* *p* < .05 (two-tailed); **p < .01 (two-tailed); ***p < .001 (two-tailed).

When comparing parental reports on younger children (*n* = 31, age 8–10 years) with those on older children (*n* = 54, age 11–17 years) in this sample, younger children reportedly had significantly more emotional symptoms, that is, *t*(83) = 2.66, *p* = .009, *d* = 0.60, 95% CI [0.15, 1.06], and hyperactivity–inattention problems, that is, *t*(83) = 2.22, *p* = .029, *d* = 0.50, 95% CI [0.05, 0.95], as well as significantly higher Total Difficulties scores, *t*(83) = 3.23, *p* = .002, *d* = 0.73, 95% CI [0.27, 1.18].

### Parental reports of own mental health, family functioning, and perceived parental control

On CORE-OM [22], 47 (81%) of the patients and six (26%) of the partners exceeded the clinical cut-off score on the Symptoms subscale, and 48 (83%) of the patients and three (13%) of the partners exceeded the cut-off on the Total Scale excluding Risk items, indicating clinical-level distress. On the HADS-A, 46 (77%) of the patients and seven (28%) of the partners had scores of 8 or above, indicating possible or probable presence of clinical-level anxiety. On the HADS Depression subscale, 44 (73%) of the patients and eight (32%) of the partners had scores of 8 or above. Regarding family functioning, FAD-GF was reportedly problematic in 36 (57%) of the 63 families. The PLOC-PPC results indicated that, in relation to their children (*n* = 87, *M* = 3.87, *SD* = 0.69), the primary informants reported significantly more perceived parental control of their children’s behaviour, *t*(155) = 3.52, *p* <.001, *d* = 0.57, 95% CI [0.24, 0.89], than did parents of the Swedish reference population (*n* = 70, *M* = 3.49, *SD* = 0.65) [14].

### Associations between study variables

Correlations between parent-reported child mental health and child, parent, and family variables revealed several significant associations (see Table III). Parents who reported lower Perceived Parental Control (PLOC-PPC) and higher levels of own anxiety (HADS-A), reported more child mental health problems (SDQ-P Total Difficulties). A significant correlation was also found between child age and reported difficulties (SDQ-P Total Difficulties), with younger children having higher levels of symptoms.

**Table III.** Pearson’s correlations between child mental health (SDQ-P Total^†^) and family functioning, perceived parental control, parent mental health, child age, and parent’s social status.

Variable	1	2	3	4	5	6	7	8	9
1. SDQ-P Total^†^	–	83	86	83	83	80	83	85	80
2. FAD-GF^‡^	−.10	–	84	84	84	81	84	83	81
3. PLOC-PPC^§^	−.60**	−.12	–	84	84	81	84	86	81
4. CORE-OM^¶^ Total excluding Risk items	.13	.34**	−.12	–	84	81	84	83	81
5. CORE-OM^¶^ Problem subscale	.17	.31**	−.12	.97**	–	81	84	83	81
6. HADS^††^ Anxiety subscale	.27*	.29**	−.36**	.72**	.76**	–	81	80	80
7. HADS^††^ Depression subscale	.20	.29**	−.18	.80**	.74**	.56**	–	83	81
8. Child age	−.42**	.03	.25*	−.04	−.06	−.08	.009	–	80
9. SS^‡‡^	−.10	.32**	.03	−.27*	−.27*	−.13	−.17	.17	–

*Note*: Values of *n* are shown above the diagonal.

† SDQ-P Total = Strength and Difficulties Questionnaire – Parent version, Total Difficulties score. ^‡^FAD-GF = Family Assessment Device, General Functioning subscale. ^§^PLOC-PPC = Parental Locus of Control Questionnaire – Control of Child’s Behaviour subscale. ^¶^CORE-OM = Clinical Outcomes in Routine Evaluation – Outcomes Measure, Total subscale excluding Risk items and Symptom subscale. ^††^HADS = Hospital Anxiety and Depression Scale. ^‡‡^SS = Social Status according to the Hollingshead Index.

* *p* < .05 (two-tailed); **p < .01 (two-tailed).

### Variables predicting child mental health

A multiple linear regression analysis was carried out to investigate whether the independent variables Perceived Parental Control (PLOC-PPC) and General Functioning (FAD-GF) could significantly predict the dependent variable child mental health problems (SDQ-P Total Difficulties), controlling for child age and gender, parent’s SS, and parental anxiety (HADS-A). The results of the regression indicated that the model explained 49% of the variance and that the model was a significant predictor of child mental health, *F*(6,71) = 13.19, *p* < .001, *R*^2^ = .53, *R*^2^_Adjusted_ = .49 (see Table IV). The independent variables child age (*b* = −0.44, *p* = .020) and Perceived Parental Control (*b* = −0.52, *p* < .001) contributed significantly to the model, while General Functioning (*b* = −0.15, *p* = .087), child gender (*b* = −1.78, *p* = .094), parent’s SS (*b* = 0.007, *p* = .834), and anxiety (*b* = 0.09, *p* = .463) did not.

**Table IV.** Multiple linear regression with child mental health (SDQ-P^†^ Total) as dependent variable.

Variable	*B*	*SE*	*β*	*t*	*p*	95% CI for *B*
Child gender^‡^	−1.78	1.05	−0.15	−1.70	.094	[−3.88, 0.31]
Child age	−0.44	0.18	−0.21	−2.38	.020**	[−0.81, −0.07]
SS^§^	0.007	0.04	0.02	0.21	.834	[−0.06, 0.08]
HADS-A^¶^	0.09	0.12	0.07	0.74	.463	[−0.14, 0.31]
PLOC-PPC^††^	−0.52	0.08	−0.58	−6.39	<.001***	[−0.69, −0.36]
FAD-GF^‡‡^	−0.15	0.08	−0.16	−1.73	.087	[−0.31, 0.02]
Constant	39.87	4.40		9.05	<.001***	[31.09, 48.65]

*Note: n* = 78. CI = confidence interval.

† SDQ-P = Strength and Difficulties Questionnaire – Parent version, Total Difficulties score. ^‡^Girls = 1, Boys = 2. ^§^SS = Social Status according to the Hollingshead Index. ^¶^HADS-A = Hospital Anxiety and Depression Scale – Anxiety subscale. ^††^PLOC-PPC = Parental Locus of Control Questionnaire – Perceived Parental Control of Child’s Behaviour subscale. ^‡‡^FAD-GF = Family Assessment Device – General Functioning subscale.

* *p* < .05 (two-tailed). **p < .01 (two-tailed). ***p < .001 (two-tailed).

### Effect of number of risk factors on child mental health

For the children experiencing 0–1 risk factors according to the constructed cumulative risk index (*n* = 16, *M* = 7.44, *SD* = 4.69), there was no significant difference in mental health compared with the Swedish population-based sample [20]; the difference was significant however, for children experiencing 2–3 risk factors (*n* = 43, *M* = 8.60, *SD* = 5.61), *t*(479) = 3.14, *p* = .002, *d* = 0.50, 95% CI [0.19, 0.82], as well as for the group experiencing 4–6 risk factors (*n* = 17, *M* = 12.41, *SD* = 7.09), *t*(452) = 5.23, *p* <.001, *d* = 1.29, 95% CI [0.80, 1.78].

There was a significant association between the number of risk factors the child experienced (i.e., 0–1, 2–3 or 4–6) and whether the child was categorised as over the cut-off on both the SDQ-P Total Difficulties and Impact scores (two-sided Fisher’s exact test, *p* = .042). It was the proportion over the cut-off in the group exposed to 4–6 risk factors that contributed the most to the overall significant result. Based on the odds ratio, the odds of being categorised as over the cut-off on both SDQ-P Total Difficulties and Impact scores were 3.40 times higher (95% CI [0.97, 11.89]) for the group experiencing 4–6 risk factors than for those experiencing 2–3 risks, and 11.67 times higher (95% CI [1.23, 110.95]) than for those experiencing 0–1 risk.

## Discussion

This study has investigated the mental health and family context of children of parents who are patients of specialised psychiatric services, diagnosed with depression, anxiety or bipolar disorder. The results show that many of these children reportedly had high levels of mental health problems and that younger children (aged 8–10 years) reportedly had more mental health problems than did older children (aged 11–17 years). Concerning family context, it was also found that a group of patients’ partners reported mental health problems at clinical levels. Furthermore, lower perceived parental control and younger child age were associated with more child mental health problems. When a cumulative index was calculated, children with 2–3 and 4–6 risk factors reportedly had increasingly more mental health problems than did children experiencing 0–1 risk factors.

According to the parental reports, the children in this study had significantly more mental health problems (SDQ-P) than did a Swedish population-based sample. This is in line with previous research findings that severe parental mental illness increases the risk of child mental health problems [3,11]. Furthermore, about one-third of the children in our sample reportedly had a high symptom load with scores above the clinical cut-off on the Total Difficulties scale (SDQ-P). This finding was also reported in an Australian study of children of patients in contact with psychiatry or welfare services [9], which found that 39% of the children reportedly had symptoms at a clinical level according to the SDQ-P Total Difficulties scale. Although our sample did not include children with their own depression or anxiety diagnoses, families in which the patient’s mental illness was in an acute phase or families in which a crisis was ongoing, the results still showed that many children had increased levels of mental health problems.

Younger children (aged 8–10 years) in our sample reportedly had significantly more mental health problems than did the older children (aged 11–17 years). The effect sizes for the differences between age groups on the SDQ-P in our sample were medium, whereas in a British population-based sample of children aged 5–15 years [30], the effect sizes of the differences between those 5–10 and 11–15 years old ranged between no difference and *d* = 0.1. Child age was also found to be a significant predictor of child mental health in the multiple regression, with younger age predicting more problems on the SDQ-P Total Difficulties scale. Previous research has noted that younger children are more negatively affected by parental mental illness, although this mainly refers to the first years of a child’s life [11,12]. A possible explanation of our results is that the younger children had been exposed to parental mental illness earlier in life, relative to the older children, and therefore were more negatively affected. The results indicate that younger children constitute a vulnerable subgroup whose needs are important to address.

Concerning the family context, the results for measures of the parent’s self-reported mental health indicate that a group of partners had symptoms in the clinical range (i.e., for CORE-OM and HADS). Previous research has shown that if both of a child’s parents have mental health problems, this increases the risk of the child developing difficulties, but if one parent can support the child, the risk can be reduced [11]. The results of our study suggest that the mental health situation of the partners could be important to address in these families, as the partner of the patient could also need support or treatment, which could in turn benefit the children. Whether the higher levels of psychological problems in the partners were due to their own difficulties or to having a partner with mental illness cannot be answered by this study.

In this study, family General Functioning (FAD-GF) was rated as problematic in over half of the families. Family functioning was, however, not found to correlate significantly with or predict child mental health in our study. The creators of the measure have concluded that families experiencing parental mental illness often report impairment in family functioning, but that this does not necessarily negatively affect the mental health of all family members [15]. One study found that child-reported, but not parent-reported, family functioning could predict depressive symptoms in the children of parents with depression [16], which could explain the results of this study, as we only have parental reports.

The participants reported high Perceived Parental Control of their child’s behaviour (PLOC-PPC); in fact, they perceived better control of troublesome child behaviours than did the reference population [14]. One possible explanation of this result is that parents with mental illness and their partners are uncomfortable acknowledging difficulties in child-rearing situations [6], leading to biased responses. In the multiple regression, PLOC-PPC was found to be a significant predictor of child mental health in our sample. Perceived parental control is arguably related to parental self-efficacy beliefs [14], and higher self-efficacy beliefs have been associated with more effective parenting styles and behaviour, whereas low parental self-efficacy as well as low perceived parental control have been associated with poorer outcomes for children [13,14]. The present results are in line with this research, but cannot say anything about causality, and bidirectional processes could be involved. However, the results indicate that there is a possibility of supporting children by supporting patients experiencing difficulties in child-rearing situations.

As the number of risk factors increased for the children in our sample, more mental health problems were reported by their parents, as also shown in previous research [17,18]. The cumulative risk index in this study included child, parent, and family risk factors as well significant predictors of child mental health found here. The factors included in such cumulative risk indexes for child mental health vary between studies, and the pattern and severity of risk factors have not been taken into account [18]. However, cumulative risk indexes have important strengths, especially when it comes to demonstrating the effect of early experiences on population health [18]. In our study, the cumulative risk index highlights the importance of considering many different risk factors when assessing the unique needs of the individual child and family, and when identifying children at high risk of developing their own mental health problems.

### Strengths and limitations

The results should be interpreted in light of important study limitations. The assessment of child mental health relied on parental reports, mainly from parents diagnosed with a severe mental illness, leading to a risk of a bias towards reporting more problems in the children [19]. However, research has shown that, despite their limitations, parental reports might be useful in predicting mental illness in children [19]. Multi-informant reports were not available in this study, but when we did have reports from both patients and partners on the same child, we compared them, and no significant differences were found.

The generalisability of the present results is subject to certain limitations. For instance, the study is based on a small group and significantly more families in which the parent had bipolar disorder agreed to participate in the study. The included sample also represents a subgroup of children of patients with the included diagnoses, since the families were recruited when they were about to receive a preventive intervention, which is not offered to all families in specialised adult psychiatry [6].

### Recommendations for future studies

Studies with larger samples and using multi-informant reports, especially including reports from the children themselves, could improve the estimation of mental health problems in this group of children. Considering the importance of age for child mental health in this study, investigations of the situation of the youngest children aged 0–7 years, who were not included in this study, are warranted. Further studies including the measure PLOC-PPC could shed light on ways to strengthen perceived parental control and on its effect on child mental health.

## Conclusion

This study has investigated children of patients with depression, anxiety or bipolar disorder in a clinical psychiatric context. The naturalistic design and ethical concerns contributed to a multi-stage process of selecting participants, possibly resulting in the children of patients of specialised psychiatric services most burdened by mental illness being less likely to be included. Nonetheless, the results underline the importance of identifying these children, as many reportedly had increased levels of mental health problems. To ensure that adequate support is initiated, several relevant risk factors need to be assessed. Furthermore, the study results indicate that the needs of younger children and of parents in their parenting role are important to address when working with parents who are patients being treated by specialised psychiatric services.
